# Predictors of Academic Achievement among Physical Education and Sports Undergraduate Students

**DOI:** 10.3390/sports6010008

**Published:** 2018-01-28

**Authors:** Ertuğrul Şahin, Resul Çekin, İlknur Yazıcılar Özçelik

**Affiliations:** 1 Faculty of Education, Department of Guidance and Psychological Counseling, Amasya University, Akbilek Neighborhood Muhsin Yazıcıoğlu Street No:7, 05100 Amasya, Turkey; 2 Faculty of Education, Department of Physical Education and Sport, Amasya University, Akbilek Neighborhood Muhsin Yazıcıoğlu Street No:7, 05100 Amasya, Turkey; resul.cekin@amasya.edu.tr (R.Ç.); ilknur.yazicilar@amasya.edu.tr (İ.Y.Ö.)

**Keywords:** physical education and sports students, academic achievement, sociodemographic variables, personality traits, core self-evaluations, achievement goal orientation

## Abstract

Although a number of studies have attempted to determine the antecedents, correlates, and consequences of students’ academic performance, there are few studies in the literature that examine the correlates of academic achievement for physical education and sports undergraduate students. The aim of this study was to investigate the relationship between the academic achievement of first-year physical education and sports students and their sociodemographics, attitudes towards the teaching profession, personality traits, and achievement goal orientations. The participants of the study consisted of 127 (67% male) physical education and sports students, ranging in age from 16 to 30 years old when they began their studies. Participants responded to a questionnaire to determine their sociodemographic characteristics, their attitudes towards the teaching profession in their high school years, their core self-evaluations, and their achievement goal orientations. Pearson correlation analysis results showed that students’ first year grade-point average (GPA) was associated with gender, high school GPA, core-self evaluations, and mastery-approach achievement goal orientation. Results of the regression analysis suggested that the three variables that predicted the students’ first year GPA were their mastery-approach scores, attitudes towards the teaching profession in high school years, and high school GPA. In order to prevent academic failure in physical education and sports students, those who do not have a mastery-approach goal orientation and who had a low high school GPA should be identified at the beginning of the academic year, so that educational interventions can be directed at these students.

## 1. Introduction

Educational institutions all around the world regard it as an important goal to improve the academic achievement of students, since academic success can significantly affect the lives of students. Academic achievement is important not only to determine how much students have learned in an educational program, but also to ascertain whether students will continue in the schools they want to attend. It also affects the results of country-wide examinations that students will take, their future careers after school, and the job opportunities they may have in the future. It is not only of concern to students, but it is also a topic that interests parents, administrators, and all other segments of society, and it is related to individuals’ future job success [1]. Thus, variables affecting academic performance have become one of the most important topics of interest among researchers.

For many years, schools have assessed student achievement either as an average of grades or scores received by a student or as a level related to reaching pre-defined learning goals. However, in order to measure students’ academic achievement, researchers evaluated the test scores obtained from nationwide exams, cumulative grade point average, and average passing scores in certain courses such as mathematics, social studies, reading, statistics, or grading and retention as an indicator of academic achievement. Academic achievement in the literature is often used interchangeably with academic performance, and academic achievement is often measured by the cumulative academic grade point average of individuals or its well-known name GPA. 

Available evidence suggests that academic performance is a complex phenomenon and should take into account different factors, including sociodemographics, personality traits, motivational factors, and attitudes. However, to date few studies have examined these factors simultaneously, especially in physical education and sports student samples. Taking into account these different factors may provide a more comprehensive understanding of the complex relationship between academic performance and other variables in physical education and sports students. Unlike other undergraduate majors in Turkey (except those who take a special talent evaluation test), students in physical education and sports departments should obtain a certain score on the general university entrance exam, and additionally, they should be successful in a special sports talent examination. Moreover, the physical education and sports department schedule includes practical lessons that will help develop a wide range of sports skills, as opposed to the numerous undergraduate programs in which theoretical knowledge and skills are taught intensively. In this context, students’ academic achievement is determined not only by their general cognitive abilities, but also partly by their sports skills. For this reason, there is a need for evidence about whether the correlates of academic achievement in different undergraduate programs can be generalized to the students of physical education and sports. The purpose of this study is to investigate the relationship between the academic achievement of freshman students of physical education and sports and sociodemographic variables, attitudes towards the teaching profession, personality traits, and achievement goal orientations.

### 1.1. Sociodemographic Variables and Academic Achievement

Sociodemographic and cognitive variables associated with academic success have become a topic of interest among researchers. The relationship between the academic achievement of undergraduate and graduate students in different departments for specific courses (e.g., social sciences, sciences, language, mathematics) or general academic achievement and variables such as gender, age, parental education level, socioeconomic level, ethnic identity, high school grade point average (GPA), university entrance examination achievement score, and marital status have been frequently investigated. Gender, high school GPA, and age were examined in accordance with the purpose of this research. 

Research investigating gender differences in academic performance generally suggests that female students tend to have higher academic achievement than male students across different education levels, albeit the effect size of these differences is low [2,3]. However, when the results of the research are examined in the context of university students, cross sectional research has shown that the majors and level of education (undergraduate, graduate) may be an important factor in the relation between academic achievement and gender [4,5,6,7,8,9,10,11]. For example, Ali et al. [5] found that gender was not related to the academic achievement of graduate students. However, studies conducted on medical, elementary education, and sports and exercise undergraduate students have shown that the academic success of girls is more likely to be higher than that of boys [7,9,11]. However, there were no significant differences in the academic achievement of girls and boys in the samples of university students in majors such as economics, business administration, finance, information technology, and arts and education [6,8,12].

Studies conducted on different samples, on the other hand, have consistently shown that previous academic performance, as measured by university entrance exam score or high school GPA, is positively correlated with university GPA [2,4,7,10,12,13]. In a meta-analysis study, Richardson et al. [2] found that high school GPA was the strongest correlated variable with university GPA when evaluated with the other traditional academic achievement correlates, such as intelligence, and Scholastic Aptitude Test (SAT). 

Lastly, although age is a variable frequently investigated as a correlate of academic achievement, there have been mixed findings regarding the relationship between age and academic performance [4,5,6,11,12]. While some research has shown that the academic achievement of young university students is higher than that of older university students [5,6], other researchers found that the academic achievement of older university students is higher than that of younger students [4,11]. Additionally, some researchers found that age did not correlate with the academic achievement of students [12].

### 1.2. Attitudes towards the Teaching Profession and Academic Achievement

While there are many definitions of attitude, the most general is a tendency to have a positive or negative reaction to a specific object, situation, institution, concept, or other people [14,15]. Attitudes towards the teaching profession may be defined as the tendency for individuals to have a positive, negative, or neutral response to the teaching profession after direct or indirect learning experiences. Attitudes towards the teaching profession may be correlated with academic performance. Recent research has shown that physical education and sports teachers’ attitudes towards the teaching profession are generally positive and that some sociodemographic variables may be related to attitudes towards the teaching profession [16,17]. However, although there are different lines of research related to attitude towards the teaching profession in the literature, there is a limited number of studies that examine the relationship between attitudes towards the teaching profession and academic achievement [7,18], and these studies generally examined how the individual’s current attitude towards the teaching profession and academic performance are related. For example, Buyukozturk [7] found that the attitude towards the teaching profession was weakly and positively associated with university GPA in a sample of elementary school education students. However, Özder et al. [18] found that attitude towards the teaching profession was not related to academic achievement in a sample of students from pre-school and primary school education majors. Inconsistent results in these studies indicate that the relationship between attitudes towards the teaching profession and academic success need to be examined in different samples. At the same time, these surveys have frequently examined the connection between present attitudes towards the teaching profession and academic performance. It is known that attitudes are generated by direct and indirect learning experiences that start from childhood and continue throughout one’s lifetime, and they are usually resistant to change [14,15]. Additionally, there is limited available research on how students’ attitudes towards the teaching profession during their high school years influence their academic success in college. Thus, the association between students’ attitudes towards the teaching profession in their high school years and academic achievement deserve further investigation. 

### 1.3. Core Self-Evaluations and Academic Achievement

One personality characteristic that may be associated with academic performance is core self-evaluations. According to Judge, Enes, Bono, and Thoresen [19], core self-evaluations are comprehensive, latent, and highly regarded personality features that are composed of (a) self-esteem: positive or negative attitudes towards one’s own self [20]; (b) self-efficacy: a person’s beliefs and expectations concerning the ability to engage in necessary and purposeful behavior [21]; (c) neuroticism: having a tendency to focus on the negative aspects of life and to have a negative cognitive structure [22]; and (d) locus of control: beliefs about the causes of events occurring in one’s life [23]. In this context, core self-beliefs include a comprehensive assessment of one’s capacity, worth, and effectiveness, as well as how one evaluates events. 

Previous research has examined the relationship between core self-evaluations and variables such as intention to leave, job satisfaction, job commitment, job performance, attitudes towards work and behaviors, work-home conflict, and so on, partly because it is a concept that first emerges in the field of industrial psychology. Surprisingly, however, there is a limited amount of research that has been conducted on student samples examining the relationship between core self-evaluations and academic achievement [24,25]. For example, in a multi-sample study, Albrecht et al. [24] found that there was no relationship between high school GPA and core self-evaluations in German high school students. In another study, Rosopa and Schroeder [25] discovered that core self-evaluations of American college students were not directly related to academic achievement; in fact, core self-evaluations are a moderating variable between cognitive skills and academic achievement, such that core self-evaluations strengthened the relationship between cognitive skills and academic success. 

These results may be due to the fact that research was carried out both at different levels of education and among individuals with different cultural backgrounds. However, the available limited research suggests further studies are required in order to better illuminate the association between core self-evaluations and academic performance in university students.

### 1.4. Achievement Goal Orientations and Academic Achievement

One of the motivational factors that can affect the academic achievement of students is their achievement goal orientations. Achievement goal orientations are defined as the aims and reasons for individuals to exhibit their behavior concerning success [26]. Historically, attempts were made to conceptualize students’ achievement goal orientations using two, three, four, and six factor models. This research examined the relationship between students’ achievement goal orientations and academic achievement using the 2 × 2 achievement goal orientation approach developed by Elliot and McGregor [27]. 

According to the 2 × 2 achievement goal orientation approach, people have four different approaches depending on the personal or societal standards they use to approach success or avoid failure. These are: mastery approach, mastery-avoidance, performance approach, and performance-avoidance. Students oriented towards the mastery-approach tend to focus on striving for success by taking into account their own personal specialization in a particular task and use their qualifications to improve proficiency by exerting a high level of effort. On the other hand, students oriented towards a mastery-avoidance approach tend to be afraid of failure, emotional, anxious, and focus on improving on their previous performance. They are more likely to have a strong desire to strive towards avoiding intrapersonal competence. 

While mastery orientations focus on intrapersonal competence, performance orientations emphasize normative/interpersonal competence. For instance, students who embrace the performance-approach compare their achievements with those of other students, express a need for success, care about proficiency, use superficial learning strategies, and need the approval of others. They define their success in relation to that of others. Finally, students who adopt the performance-avoidance approach tend to use superficial learning strategies. They tend to have a strong belief that people should strive to avoid doing worse than others [27].

Studies have shown that different achievement goal orientations are related to academic achievement in different directions. For example, while mastery achievement goal orientation and performance achievement goal orientation were positively correlated with academic achievement [28], performance-avoidance was negatively correlated with academic achievement [29]. 

### 1.5. Research Hypotheses

Based on the above mentioned limited studies on physical education and sports undergraduate students, and taking into account the Turkish cultural context, we formulated the following research hypotheses:

**Hypothesis** **1.** *The academic achievement of female physical education sports students will be higher than that of male physical education sports students*0.

**Hypothesis** **2.** *High school GPA will positively correlate with first year GPA scores in physical education and sports students*0.

**Hypothesis** **3.** *The age at which a student begins university will not correlate with first year GPA scores in physical education and sports students*0.

**Hypothesis** **4.** *Attitudes towards the teaching profession in high school years will positively correlate with first year GPA scores in physical education and sports students*0.

**Hypothesis** **5.** *Core self-evaluations will be positively associated with first year GPA scores in physical education and sports students*0.

**Hypothesis** **6.** *The mastery-approach will be positively related to first year GPA scores in physical education and sports students*0.

**Hypothesis** **7.** *Mastery-avoidance will not be correlated with first year GPA scores in physical education and sports students*0.

**Hypothesis** **8.** *The performance-approach will be positively correlated with first year GPA scores in physical education and sports students*0.

**Hypothesis** **9.** *Performance-avoidance will not be associated with first year GPA scores in physical education and sports students*0.

## 2. Methods

### 2.1. Participants

The study participants consisted of 152 students who were part of the physical education and sports teaching department of a university in the Central Black Sea region of Turkey. They were selected using convenience sampling. However, 25 students who did not correctly answer attention check items in the questionnaire (for example: Please answer this question by choosing option number two, “disagree.”) have been removed from the dataset. The students were given course credit for participating in the research. The participants were 42 females (33%) and 85 males (67%). The age at which they began university ranged from 16 to 30, with an average age of 19.22 (*SD*: 1.80). 

### 2.2. Scales

Personal Information Form: Participants were asked to give information about their gender, age, and grade level in this form. In order to calculate the age at which students began university, we subtracted their years of study from their age.

Previous Attitudes towards Teaching Profession: The Attitudes towards Teaching Profession Scale (ATTPS) developed in the context of the Turkish culture was used to measure the attitudes of the participants towards the teaching profession. It was developed by Üstüner [30] in order to measure the attitudes of university students studying in teaching programs towards the teaching profession. The scale is a one-dimensional structure consisting of 34 items, and 10 items are reverse coded. Participants indicate the degree of agreement with each item of the scale by marking one of the options ranging from *Strongly Disagree* (1) to *Strongly Agree* (5). In this study, only 17 items of the scale were used in order to reduce the participants’ burden. At the same time, since their previous attitude towards teaching also needed to be determined, the expressions in the scale were modified to reflect the past, and the statement “Pretend that you are in high school” was added to the instructions. For example, the item that read “I sympathize with teachers” was changed to “I used to sympathize with teachers”. Since only 17 items were used in the scale, item analysis and exploratory factor analysis were performed to uncover the reliability and underlying factor structure of the scale, respectively. Five items (2, 4, 12, 13, 14) with item total correlations below 0.45 were excluded from the analysis. Table 1 shows the reliability analysis results. As seen in Table 1, the item-total correlations of the remaining 12 items ranged from 0.46 (item 10) to 0.69 (item 8), with a Cronbach alpha internal consistency coefficient of 0.86. The mean inter-item correlations were 0.35. As a general rule, researchers indicate that measurement tools with a Cronbach alpha internal consistency coefficient of 0.70 and above can be used for screening and research purposes [31,32]. Additionally, the mean inter-item correlations values were within the acceptable range that is in the range of 0.15 to 0.50, as suggested by Clark and Watson [33]. These findings indicate that the scale has sufficient reliability. In order to determine underlying dimensions of scale items, exploratory factor analysis was performed in the second stage. When determining the number of factors to retain in exploratory factor analysis, the eigenvalues greater than one rule, the scree plot graph, the parallel analysis, and Velicer’s Minimum Partial Test (Map), as well as the interpretability of the obtained factors, were used as criteria. Furthermore, in order to determine which item belonged to which factor, we used item factor loading values of 0.45 or higher, and there was to be a difference of at least 0.10 or higher between corresponding salient item factor loadings and other factor loadings. Preliminary analysis was performed to examine the suitability of the ATTPS items’ correlation matrix for conducting factor analysis. 

Bartlett’s Test of Sphericity was significant (*x*^2^ (66) = 572.36, *p* < 0.001), and the Kaiser-Meyer-Olkin sample adequacy coefficient was 0.84, well above the suggested minimum value for conducting a factor analysis [34]. These findings suggest that the obtained data correlation matrix was appropriate for performing factor analysis. The eigenvalue greater than unity rule found that the correlation matrix of the ATTPS items was composed of three factors with an eigenvalue higher than one. Specifically, the eigenvalues of the scale were found to be 4.86, 1.46, and 1.08, respectively. The scree plot graph showed a clear, broken-stick pattern in one factor. Similarly, Velicer’s MAP [35] test minimum partial correlation value was 0.037 and revealed that the factor structure of the scale should consist of only one factor. Finally, Horn’s [36] parallel analysis test results demonstrated that only the first factor’s eigenvalue was above the mean eigenvalues obtained from 500 randomly generated samples; in other words, the scale should consist of only one factor. Research has indicated that the use of parallel analysis and Velicer’s MAP test give more reliable results than other retaining techniques used for assessing the number of factors in exploratory factor analysis [37,38]. Since multiple factor retention decision criteria suggested extracting different numbers of factors, we performed two separate factor analyses using one and three factor forced solutions with Principal Axis Factoring. When we extracted three factors, the oblique Promax rotation (κ = 4) was used. Principal Axis Factoring with oblique Promax rotation for three factor solution suggested that factor loading of many items was below the 0.45, and three items loaded on more than one factor (in other words, they cross-loaded). Moreover, no interpretable factor structure emerged. Thus, we decided to extract one factor solution for ATTPS items, in line with the scree test, parallel analysis, and Velicer’s MAP test, as well as emerging exploratory factor analysis results. Table 1 shows forced one factor solution using Principal axis factoring. As shown in Table 1, the item factor loadings of the scale ranged from 0.48 to 0.74. Moreover, forced single factor solution yielded an interpretable factor structure and good coherence between items. Possible scores ranged from 12 to 60, and higher scores indicate more positive attitudes towards teaching in high school years. 

Core Self-Evaluations Scale (CSES): The CSES developed by Judge et al. [19] was used to measure the coreself-evaluations of participants. The scale’s psychometric properties have been examined by different researchers for the Turkish culture. It has been found to be a valid and reliable scale that can be used in the assessment of university students and adults [39,40]. The scale consists of 12 items, and the participants indicate their degree of agreement with each item by marking one of the options ranging from *Strongly disagree* (1) to *Strongly agree* (5). Six items in the scale are reverse coded such that higher scores reflect more positive core self-evaluations. Possible scores range from 12 to 60. The internal consistency coefficient of the CSES calculated in this study was 0.73.

Revised 2 × 2 Achievement Goal Orientation Scale (AGOS-R): This scale was developed by Elliot and Murayama [41]. The Turkish language adaptation, validation, and reliability study was conducted by Arslan and Akın [42]. The scale aims to measure students’ achievement goal orientations in four dimensions. Despite the development of different models and scales [43] to measure achievement goal orientations up to this point in time, this scale was still the most recently translated scale that could be used when this research was carried out. The scale consists of 12 items, and participants rate each item on a five-point Likert type scale ranging from *Strongly disagree* (1) to *Strongly agree* (5). The mastery-approach, mastery-avoidance, performance-approach, and performance-avoidance achievement goal orientation are measured by three items. Scores that can be obtained from each dimension range from 5 to 15. Higher scores indicate higher achievement goal orientation for each subscale. The internal consistency coefficient of the subscales calculated in this study were 0.87 for the mastery-approach subscale, 0.65 for the mastery-avoidance subscale, 0.88 for the performance-approach subscale, and 0.82 for the performance-avoidance subscale. 

### 2.3. Procedure

The data used in the study were collected during the 2015–2016 academic year. Information on first year GPA scores and high school GPA scores were obtained from the Department of Student Affairs after the necessary permission was received from the students at the end of the academic year 2015–2016. The students answered the surveys individually or as a group under the supervision of the second and third researcher at the beginning or end of their courses during regular class hours. Students were given a course credit for responding to the questionnaires. Ethical considerations of the study were clearly assured before administration of the questionnaires. The students were informed about voluntary participation, confidentiality, anonymity, and that they could withdraw from the study at any time without penalty. All students voluntarily answered the surveys. Questionnaires completed by the students were checked by the researchers to determine if there were any incomplete responses, and participants who did not provide complete responses to questionnaire items were asked to provide answers to those items. Completion of the questionnaires required approximately 20 min. 

### 2.4. Statistical Analysis

Statistical analysis was performed in SPSS 23, Lisrel 9.3, and Factor 9.3 [44] programs. Prior to performing the analysis, the accuracy of the data, missing values, outliers, and the assumptions of statistical analyses were examined. First, the frequency distributions of the data, as well as the minimum and maximum values, were checked. All values were within the acceptable range. In the second stage, the missing values were checked, and no missing value was found in the data. In the third stage, outlier values were checked, because the planned correlation analysis and regression analysis were sensitive to outliers [45,46]. In this context, participants’ scores from continuous variables were converted to standardized z-scores, and univariate outliers were examined based on the suggestion of Tabachnick and Fidell [45]. Univariate outliers observed in the study are as follows: one in the first year GPA, one in the age at which students began university, one in the core self-evaluation scale, and one in the mastery-approach scale. Given the limited sample size in our study, these univariate outliers were winsorized as suggested by Tabachnick and Fidell [45]. In other words, the scores of the participants with the outlier values were changed according to the scores of the participants with the highest or lowest value that were not outliers in the distribution plus one or minus one. For example, the outlier with a mastery-approach score of 3 was replaced by the next possible lowest value of 4 in the distribution. The above procedure was applied to all univariate outliers. In order to detect multivariate outliers in the regression analysis, Cook’s distances were calculated by using first year GPA scores as the dependent variable and all other variables as independents, and eight influential data points were detected and excluded from the dataset using the cut-off scores above 4/*N* in which *N* is the sample size of the study for Cook’s distance. For this reason, analyses reported in the results section were based on 119 participants.

Descriptive statistics were used to determine the general characteristics of the research sample. An independent samples *t*-test was used to compare first year GPA scores between males and females. Pearson correlation analysis was employed to ascertain the relationships between the study variables. A standard multiple regression was performed to assess the ability of sociodemographic variables, attitudes towards the teaching profession, core self-evaluations, and achievement goal orientations to predict first year GPA scores. The forced entry method was used in multiple regression analysis. In this method, all predictors were simultaneously entered into the regression equation [47]. Correlation analyses were reported with bias corrected and accelerated 95% confidence interval based on a 10,000 bootstrap sample. Preliminary analyses were conducted to ensure no violation of the assumptions of normality, linearity, multicollinearity, and homoscedasticity. The significance level was set at *p* < 0.05 for all statistical analyses.

## 3. Results

Table 2 shows the descriptive statistics for the variables of interest. As shown in Table 2, participants’ first year GPA and high school GPA were at the medium level. Specifically, students’ first year GPA scores ranged from 46.28 to 83.66, and the average was 69.56 (*SD*: 7.14). Moreover, students’ high school GPA scores ranged from 50 to 90 and the mean GPA was 67.04 (*SD*: 7.84).

Closer examination of Table 2 also suggests that participants’ attitudes towards the teaching profession in high school were generally positive. Similarly, the participants’ core self-evaluations were also high. The mean mastery approach orientation scores were higher than the mean mastery avoidance, performance-approach and performance-avoidance goal orientation scores.

In order to be able to examine gender differences in first year GPA scores, an independent samples *t*-test was performed, and the results are reported in Table 30. As seen in Table 3, there was a significant difference between groups (*t*(117) = 2.15, *p* < 0.05, *r*^2^ = 0.04), such that female students (*M* = 71.50) had significantly higher first year GPA scores than male students (*M* = 68.57) though the difference was small. 

Table 4 below shows the correlation coefficients between the first year GPA scores and the sociodemographic characteristics, attitudes towards the teaching profession, core self-evaluations, and achievement goal orientations with bias corrected and accelerated confidence intervals based on resampling with replacement from the original dataset. As seen in Table 4, there was no significant relationship between the first year GPA scores and the age at which students began university (*r* = 0.00, 95% [−0.19, 0.18], *p* > 0.05), first year GPA scores and attitudes towards to the teaching profession in the past (*r* = −0.09, 95% [−0.25, 0.09], *p* > 0.05), first year GPA scores and mastery avoidance achievement goal orientation scores (*r* = 0.08, 95% [−0.09, 0.25], *p* > 0.05), first year GPA scores and performance-approach goal orientation scores (*r* = 0.17, 95% [0.01, 0.32], *p* > 0.05), or first year GPA scores and performance-avoidance goal orientation scores (*r* = 0.00, 95% [−0.17, 0.17], *p* > 0.05). However, first year GPA scores were weakly and positively correlated with high school GPA scores (*r* = 0.24, 95% [0.08, 0.39], *p* < 0.01), core self-evaluations scores (*r* = 0.18, 95% [0.03, 0.33], *p* < 0.05), and mastery-approach scores (*r* = 0.24, 95% [0.07, 0.39], *p* < 0.05), but weakly and negatively associated with gender (*r* = −0.20, 95% [−0.34, −0.03], *p* < 0.05). Moreover, as seen in Table 4, the 95% confidence intervals for all variables were quite large indicating that the true correlation values can vary greatly in the population. 

In order to be able to determine the variables that predict first year GPA scores, a multiple regression analysis was performed using the forced entry method. The change statistics for the regression analysis are shown in Table 5, and the multiple regression analysis results are shown in Table 60.

As shown in Table 5, the explained variance by model (Model 1) was statistically significant (*F* (9, 109) = 3.00, *p* < 0.01, Δ*R*^2^ = 0.20) and accounted for approximately 20% of the changes in first year GPA scores. Examination of the individual contribution of independent variables in Table 6 suggests that gender (β = −0.12, *t* (109) = −1.32, *p* > 0.05), age at which students began university (β = −0.05, *t*(109) = −0.52, *p* > 0.05), core self-evaluations (β = 0.13, *t* (109) = 1.43, *p* > 0.05), mastery-avoidance (β = −0.09, *t* (109) = −0.08, *p* > 0.05), performance-approach (β = 0.14, *t* (109) = 1.21, *p* > 0.05), and performance-avoidance (β = −0.13, *t* (109) = −1.28, *p* > 0.05) goal orientation scores were not associated with first year GPA scores. However, high school GPA scores (β = 0.19, *t* (109) = 2.27, *p* < 0.05), attitudes towards the teaching profession in high school years scores (β = −0.23, *t* (109) = −2.34, *p* < 0.05), and mastery-approach achievement goal orientation scores (β = 0.29, *t* (109) = 3.08, *p* < 0.05) were significant predictors of first year GPA scores. While high school GPA scores and mastery achievement goal orientation scores weakly and positively correlated with first year GPA scores, attitudes towards the teaching profession in high school years scores were weakly and negatively correlated with first year GPA scores. 

## 4. Discussion

The determining factors such as sociodemographic variables, personality traits, and academic and educational factors related to university students’ academic achievement have been a topic of interest to many researchers, educators, and policy makers. Developing strategies to increase the academic achievement of students who attend different university departments can help to facilitate educational interventions and increase the academic achievement of students who may face the risk of academic failure. In this study, the relationship between academic achievement and sociodemographic variables, core self-evaluations, and achievement goal orientations of physical education and sports students, who have been considered in a limited amount of research, were examined. 

As a result of this research, consistent with our first research hypothesis, it was found that female students’ first year GPA scores were significantly higher than those of male students. However, the difference between females and males in terms of GPA has a small effect size. These results are in line with limited empirical evidence related to sports and exercise students [11], showing that female physical education and sports students are more likely to have higher academic performance than male students, as well as meta-analysis studies showing that the effect size of the difference between male and female GPA is low [2,3]. A number of sociocultural theories have been developed to explain academic achievement differences between female and male students. One of these theories is the Expectation-Value theory [48]. According to this theory, success behaviors can be predicted by future expectations of success and importance given to a particular task. Therefore, from the point of view of the Expectation-Value theory, a student may not be motivated enough to work hard if s/he has a low expectation of success and thinks that the courses s/he learned are very small value in the future. When assessed from the point of view of this model, female students may generally have higher expectations and values than men, which can positively affect academic performance. Our findings also extend and add some new insights into the complex relationship between academic achievement and gender, such that, although the academic achievement of female students was higher than that of male students at the end of the first year of university, when considered with other sociodemographic, personality, and motivational factors, as well as the unique Turkish cultural context, gender did not predict first year GPA scores.

Social role models that try to explain gender differences in psychological traits [49,50] suggest that cultural values are one of the most important factors that determine how people think, feel, and behave in certain situations, and that human behavior cannot be considered independently of social, cultural, and contextual factors. When it is considered in terms of the Turkish cultural context, Turkish society has a high expectation of academic achievement from students, since they need to deal with a challenging and competitive environment (to be successful at examinations) and to be well-prepared for prospective opportunities. 

Consistent with our predictions, results of this study suggested that high school GPA was related to academic performance, and it was an important predictor of the academic success of first year university students. These findings are consistent with the results of previous research showing that high school GPA is related to university academic achievement in different samples [2,4,7,10,12,13]. Results of the present study also extend previous research, showing that high school GPA may be a correlate for students in physical education and sports departments.

Study results have shown that the age at which students begin university was not related to academic achievement. These findings support our research hypothesis and are similar to some previous research findings in the literature that indicate that age is not associated with academic achievement in first year students [12]. However, in the literature, there are different conclusions about how age influences academic achievement [4,5,6,11]. Closer examination of these studies reveals significant methodological differences. For example, Sheard [11] included age categorized in two groups (under 21 years old, 21 years old and over) in the study sample and analyzed age as a nominal variable in order to investigate the relationship between age and academic achievement for students who study in sports-related departments. We used age as a continuous variable, and the analyses were carried out accordingly. 

In addition, the age groups examined in these studies show significant range differences. For example, while the relation between age and academic achievement was examined with students whose age range was from 16 to 30 in this study, AL-Mutairi [6] examined the relationship between age and academic performance by dividing participants into two groups of less than 30 years old and over 30 years of age. Similarly, Alhajraf and Alasfour [4] examined six categorizations of age, ranging from 18–20 years to 29 years and older. Lastly, research on the effects of age on academic performance has also compared academic success at different times. For instance, in this study and in Duff [12], the relationship between age and first year university students’ academic achievement was investigated, whereas the above mentioned studies [4,5,6,11] employed academic success of the students at the end of the educational process as a criterion. 

For this reason, the methodological differences in these studies are not sufficient to determine in which conditions and how age has an effect on the academic performance of students who study in different departments. However, the findings of this research provide evidence that age is at least not related to the academic performance of the freshman students in the physical education and sports department. Furthermore, there is a need for studies on how age affects academic achievement at different grade levels and in different departments.

According to Ajzen [14,51], attitude is one of the important factors that determines the behavior of the individual. For this reason, past and present attitudes of prospective teachers may affect their academic performance. In this context, the relationship between attitudes towards the teaching profession in the past and subsequent academic performance, which is seldom investigated in the literature, has been examined in this research and found to be negatively correlated with subsequent academic achievement. In other words, students who have positive attitudes towards the teaching profession in the past are more likely to have lower first year GPA scores. These findings are unexpected and inconsistent with our research hypothesis and previous empirical evidence showing that current attitudes towards the teaching profession are positively correlated with academic performance [7] or are not related to current attitudes towards the teaching profession [18]. Although it is difficult to explain these findings, one possible explanation may be the sample characteristics of the study.

Results from the current study have shown that core self-evaluations are not related to academic success. These findings are inconsistent with our expectations but similar to previous evidence from high school students, showing that core self-evaluations are not related to academic achievement [24], and we extend these findings to undergraduate students in physical education and sports education. According to Rosopa and Schroeder [25], although core self-evaluations are not directly related to the academic achievement of university students, core self-evaluations are a moderator variable that reinforces the relationship between cognitive skills and academic performance. However, considering that there is a limited number of studies that examine the relationship between core self-evaluations and academic achievement, there is a need for further research to determine the relationship between core self-evaluations and academic performance in different sample groups.

Finally, results indicated that mastery achievement goal orientations were positively correlated with the academic achievement of the students of the physical education and sports department and were a significant predictor of their academic success. These findings are consistent with our research hypothesis and similar to those of previous evidence showing a positive relationship between mastery achievement goal orientations and academic achievement [28]. Students with mastery achievement orientation are more likely to be involved in the learning process, personal development, how the learning process occurs, and to put in more effort; that is, they usually focus on *learning how to learn*0. In this context, the students with a mastery-approach orientation are expected to be more successful [41]. 

This research has some limitations. First, it was carried out on a limited number of first year students in the physical education and sports departments of a small public university located in the Central Black Sea region of Turkey. For this reason, the external validity of this study is low. Future research should examine the academic achievement of students at different grade levels, as well as in different departments. Second, a cross-sectional research design was used in this study. Although the cross-sectional study provides information about the current situation in the studied sample, causality cannot be established between the findings, and it can only give information about the existence of possible risk or protective factors. Thus, in future studies, it may be useful to carry out longitudinal studies. Third, in this study, participants were asked to evaluate their attitudes towards teaching in the past. Participants’ evaluations may not exactly reflect their attitudes towards the teaching profession in high school because of their difficulty of remembering, as well as changes in attitudes over time. 

## 5. Conclusions

Correlates of academic achievement in freshman students of physical education and sports departments were examined in this study, and it was found that mastery-approach achievement goal orientation and high school GPA are related to academic achievement. In this context, considering previous academic achievement as a variable that could inform the future academic achievement of students, early academic intervention programs should be developed, taking into account previous academic achievement when identifying students at risk of academic failure. In order to prevent academic failure of students in physical education and sports departments, screening studies should be carried out at the beginning of the academic year so that students who do not have a mastery achievement orientation and who have a low high school GPA can be determined. Subsequently, the necessary educational interventions can be applied to those students. 

## Figures and Tables

**Table 1:** ATTPS Exploratory Factor Analysis and Reliability Analysis Results.

	*λ*	*Initial h*^2^	*Extraction h*^2^	*r*
ATTPS8	0.74	0.58	0.55	0.69
ATTPS7	0.70	0.52	0.49	0.62
ATTPS9	0.68	0.53	0.47	0.62
ATTPS5	0.65	0.50	0.43	0.59
ATTPS16	0.61	0.56	0.37	0.59
ATTPS1	0.61	0.42	0.37	0.58
ATTPS 6	0.54	0.45	0.30	0.49
ATTPS17	0.54	0.38	0.29	0.48
ATTPS3	0.51	0.39	0.26	0.48
ATTPS15	0.51	0.37	0.26	0.46
ATTPS11	0.50	0.37	0.25	0.47
ATTPS10	0.48	0.37	0.23	0.46
Eigenvalue	4.86			
Explained Variance (%)	40.53			
Mean Inter-Item Correlations				0.35
Cronbach alpha (α)				0.86
95% CI Cronbach alpha				0.82–0.89

Note: ATTPS: Attitudes Towards Teaching Profession Scale, *λ* = Item Factor Loading, *h*^2^ = Communality, *r* = Item-total Correlation, *n* = 127.

**Table 2:** Descriptive Statistics of the Study Variables.

	*M*	*SD*	*Min.*	*Max.*	*Possible Score*	*Skewness*	*Kurtosis*
1. First year GPA	69.56	7.14	46.28	83.66	0–100	−0.70	0.74
2. Gender	1.66	0.47	1	2	1–2	−0.70	−1.53
3. Age at time of entering university	19.20	1.56	16	25	-	0.98	1.70
4. High school GPA	67.04	7.84	50	90	0–100	−0.06	−0.22
5. Attitude	49.86	7.80	25	60	12–60	−1.05	1.34
6. Core self-evaluations	42.94	6.00	23	56	12–60	−0.26	0.53
7. Mastery-approach	11.90	2.16	5	15	3–15	−0.74	0.73
8. Mastery-avoidance	11.19	2.19	5	15	3–15	−0.47	−0.14
9. Performance-approach	11.58	2.74	3	15	3–15	−0.80	0.24
10. Performance-avoidance	11.50	3.09	3	15	3–15	−0.99	0.38

Note: *N* = 119.

**Table 3:** Results of Independent Samples *t*-test.

	*n*	*M*	*SD*	*df*	*t*	*p*
Female	40	71.50	5.83	117	2.15	0.034 *
Male	79	68.57	7.56			

Note: * *p* < 0.05.

**Table 4:** Correlations Among Study Variables.

	1	2	3	4	5	6	7	8	9	10
1. First Year GPA	-									
2. Gender	−0.20 *									
95% CI	−0.34, −0.03	-								
3.Beginning university age	0.00	0.28 **								
95% CI	−0.19, 0.18	0.13, 0.42	-							
4. High School GPA	0.24 **	−0.22	−0.17	-						
95%CI	0.08, 0.39	−0.38, −0.04	−0.35, 0.00							
5. Attitudes towards the teaching profession	−0.09	−0.06	−0.12	−0.06	-					
95%CI	−0.25, 0.09	−0.24, 0.13	−0.34, 0.10	−0.24, 0.13						
6. Core self-evaluations	0.18 *	0.06	0.18	0.00	0.10	-				
95%CI	0.03, 0.33	−0.12, 0.26	0.02, 0.32	−0.19, 0.17	−0.10, 0.31					
7. Mastery-approach	0.24 *	−0.09	0.28 **	−0.01	0.34 **	0.27 **	-			
95%CI	0.07, 0.39	−0.28, 0.10	0.09, 0.44	−0.22, 0.18	0.17, 0.52	0.08, 0.45				
8. Mastery-avoidance	0.08	0.01	0.18	−0.15	0.13	0.21 *	0.61 **	-		
95%CI	−0.09, 0.25	−0.16, 0.19	−0.01, 0.34	−0.34, 0.04	−0.06, 0.33	0.01, −0.39	0.44, 0.74			
9. Performance-approach	0.17	−0.13	0.13	−0.11	0.29 **	0.23 *	0.55 **	0.34 **	-	
95%CI	0.01, 0.32	−0.31, 0.06	−0.05, 0.29	−0.27, 0.06	0.13, 0.45	0.06, 0.39	0.36, 0.72	0.13, 0.52		
10. Performance-avoidance	0.00	−0.07	0.08	0.00	0.05	−0.01	27 **	0.10	0.45 **	-
95%CI	−0.17, 0.17	−0.25, 0.11	−0.16, 0.31	−0.18, 0.19	−0.13, 0.23	−0.18, 0.18	0.06, 0.47	−0.11, 0.30	0.24, 0.63	

Note: Gender: 1: Female, 2: Male, * *p* < 0.05, ** *p* < 0.01, Bootstrap results are based on 10,000 bootstrap samples, *N* = 119.

**Table 5:** Change Statistics for First Year University GPA score.

Model	*R*	*R*^2^	Adj *R*^2^	*SE Est.*	Change Statistics				
					Δ*R*^2^	Δ*F*	*df*_1_	*df*_2_	*p*
Model 1	0.45	0.20	0.13	6.65	0.20	3.00	9	109	0.003 *

Note: * *p* < 0.01.

**Table 6:** Results of Multiple Regression Analysis.

Model 1	Unstandardized Coefficients		95% Confidence Interval *B*	Standardized Coefficients	*t*	*p*
	*B*	*SE*	Lower-Upper	β		
Constant	59.78	12.33	35.35, 84.20		4.85	0.001 **
Gender	−1.85	1.40	−4.63, 0.94	−0.12	−1.32	0.191
Beginning university age	−0.23	0.45	−1.12, 0.66	−0.05	−0.52	0.606
High School GPA	0.18	0.08	0.01, 0.34	0.19	2.13	0.036 *
Attitudes	−0.21	0.09	−0.38, −0.03	−0.23	−2.34	0.021 *
Core self-evaluations	0.16	0.11	−0.06, 0.37	0.13	1.43	0.156
Mastery-approach	0.96	0.44	0.08, 1.84	0.29	2.17	0.032 *
Mastery-avoidance	−0.29	0.36	−1.01, 0.43	−0.09	−0.80	0.426
Performance-approach	0.36	0.30	−0.23, 0.96	0.14	1.21	0.228
Performance-avoidance	−0.29	0.23	−0.74, 0.16	−0.13	−1.28	0.202

Note: Gender 1: Female, 2: Male, Attitudes: Attitudes towards the teaching profession, * *p* < 0.05, ** *p* < 0.001.

## Supplementary Materials

The following are available online at http://www.mdpi.com/2075-4663/6/1/8/s1, the questionnaire.
