# Supplementary material for: Predictors of Academic Achievement among Physical Education and Sports Undergraduate Students
Source: Sports (Basel). 2018 Jan 28;6(1):8. doi: 10.3390/sports6010008 (PMC5969202; doi:10.3390/sports6010008)
Supplement: Supplementary file 1 [file sports-06-00008-s001.pdf]

PIF

STUDENT NUMBER:

1. What is your gender?: Female ( ) Male ( )

2. What is your age?: ……………. (Please write your age in the blanks.)

3. What is your grade level? First year university student ( ) Second year university student ( ) Third year university student ( ) Fourth year university student ( )

ATTPS

Some statements related to the teaching profession are given below. These statements aim to determine your thoughts in the past towards the teaching profession. Thus, assess every item CONSIDERING YOUR HIGH SCHOOL YEARS, and mark 1 of the 5 options that is most appropriate for you. **For some items, you are expected to respond specifically. Please complete these items accordingly to ensure the assessment process of scale.** Please, answer each item and do not leave a question blank.

NO		**Strongly Disagree**	**Disagree**	**Neither Agree Nor Disagree**	**Agree**	**Strongly Agree**
**1**	Even the idea of being a teacher was very appealing for me.					
**2**	I thought teaching as a boring profession.					
**3**	I used to ascribe the teaching profession to me.					
**4**	I used to think that teaching profession was not for me.					
**5**	I used to believe in that I would be successful in this profession.					
**6**	I wanted to do teaching even under hard circumstances.					
**7**	I think I have a peculiar ability in teaching profession.					
**8**	I used to think that teaching profession would provide me with opportunities to produce and create something.					
**9**	I used to think that I could do teaching in a professional way.					
**10**	I used to sympathize with teachers.					
**11**	Working conditions of teaching profession were appealing for me.					
**12**	I used to discuss education, learning, teaching and teaching profession with my friends.					
**13**	I used to believe in that teaching profession would bring me respect in society.					
**14**	I used to chat with teachers.					
**15**	I used to believe that when I became a teacher, people would value me.					
**16**	The permanence of teaching profession used to reassure me.					
**17**	I used to rely on myself in meeting the necessities of teaching profession.					

AGOS-R

Below are statements that reflect the thoughts of individuals regarding their lessons. Read every expression. Then, indicate your thoughts and feelings about that expression by placing a (X) in the box where you think it best suits you. **Some of the items will ask you to answer them in a particular way. Please answer these items as they want to answer.** Mark the answer that reflects your thoughts and feelings in general, without spending too much time on scale. Thank you very much for your contribution

NO		**Strongly Disagree**	**Disagree**	**Neither Agree Nor Disagree**	**Agree**	**Strongly Agree**
**1**	My aim is to completely master the material presented in this class.					
**2**	I am striving to do well compared to other students.					
**3**	My goal is to learn as much as possible.					
**4**	My aim is to perform well relative to other students.					
**5**	My aim is to avoid learning less than I possibly could.					
**6**	My goal is to avoid performing poorly compared to others.					
**7**	I am striving to understand the content of this course as thoroughly as possible.					
**8**	My goal is to perform better than the other students.					
**9**	My goal is to avoid learning less than it is possible to learn.					
**10**	I am striving to avoid performing worse than others.					
**11**	I am striving to avoid an incomplete understanding of the course material.					
**12**	My aim is to avoid doing worse than other students.					

CSES

Instructions: Below are several statements about you with which you may agree or disagree. Using the response scale below, indicate your agreement or disagreement with each item by placing the appropriate number on the line preceding that item.

1	2	3	4	5
Strongly Disagree	Disagree	Neutral	Agree	Strongly Agree

1. _____ I am confident I get the success I deserve in life.
2. _____ Sometimes I feel depressed.
3. _____ When I try, I generally succeed.
4. _____ Sometimes when I fail I feel worthless.
5. _____ I complete tasks successfully.
6. _____ Sometimes, I do not feel in control of my work.
7. _____ Overall, I am satisfied with myself.
8. _____ I am filled with doubts about my competence.
9. _____ I determine what will happen in my life.
10. _____ I do not feel in control of my success in my career.
11. _____ I am capable of coping with most of my problems.
12. _____ There are times when things look pretty bleak and hopeless to me.

Note: **Attention check items in the questionnaire were removed.**
